# The factors that influence care home residents’ and families’ engagement with decision-making about their care and support: an integrative review of the literature

**DOI:** 10.1186/s12877-022-03503-8

**Published:** 2022-11-17

**Authors:** Brighide Lynch, Assumpta A. Ryan, Marie O’Neill, Sarah Penney

**Affiliations:** School of Nursing and Institute of Nursing and Health Research, Ulster University, Magee Campus, Northland Road, BT48 7JL Londonderry, UK

**Keywords:** Care home, Resident, Families, Shared decision-making, Engagement, Care, Support

## Abstract

**Background:**

As care homes play an important role in the lives of an increasing number of older people, it is pivotal to understand how residents’ and their families engage in decision-making about their care and support. Internationally, there is an increasing emphasis in long-term care settings on the right of residents to be actively involved in all aspects of decision-making about their care and support. However, the steps necessary to achieving a culture of shared decision-making in long-term care settings remain unclear. The aim of this literature review is to summarise what is known in the literature about the factors that influence care home residents’ and families’ engagement with decision-making about their care and support.

**Methods:**

An integrative literature reviews was carried out, guided by the methodological framework proposed by Whittemore and Knafl (2005). CINAHL, Medline Ovid and ProQuest Health and Medical databases were searched for relevant articles from 2011 to 2021. A three-step method was used, including the use of reference and citation management software to manage search results and identify duplicate citations. Abstracts and full texts were reviewed by two reviewers. Details of the selected articles were then extracted using the Data Extraction Form.

**Results:**

In total, 913 articles were located and 22 studies were included in the final analysis. The thematic analysis identified three main themes that illustrate the complexities of shared decision-making in care homes: (a) a positive culture of collaborative and reciprocal relationships; (b) a willingness to engage and a willingness to become engaged; and (c) communicating with intent to share and support rather than inform and direct.

**Conclusion:**

The implementation of shared decision-making in care homes is highly dependent on the support and nurturing of collaborative and reciprocal relationships between residents, families, and staff. Part of this process includes ascertaining the willingness of residents and families to become engaged in shared decision-making. Communication skills training for staff and guided approaches that view decision-making as a supportive process rather than a once off event are essential prerequisites for implementation.

**Supplementary Information:**

The online version contains supplementary material available at 10.1186/s12877-022-03503-8.

## Background

As the population of older people continues to rise globally and the incidence of long-term conditions such as dementia increase, care homes progressively play a more important role in the lives of older people [1]. It is therefore pivotal to understand how care home residents’ and their families engage in decision-making about their care and support, and how care home staff support residents to express their preferences and wishes about life in the care home. In the United Kingdom (UK) an estimated 426,000 older people live in approximately 18,000 care homes [2]. On 1st October 2018, there were 16,007 beds in all registered nursing and residential care homes across Northern Ireland. This represents a 4% increase in the total number of beds in the sector over a ten-year period [3]. Older people are entering care homes with more complex conditions and higher levels of physical and cognitive impairment than previous years. During the recent pandemic, almost one in six residents in care homes had confirmed COVID 19 [4]. The combination of these multiple and complex needs, along with the experience of moving to the care home, present older people, and their relatives with significant challenges [5]. Research by Ryan and McKenna, [6], and O’Neill et al. (2020b) [7] reveals that communication and a caring partnership between residents, families and care home staff are crucial and need to be developed to maximise the quality of life of older people in care homes. Shared decision-making is considered the pinnacle of relationship-centred care [8, 9] and is one of the eight best practice themes that supports the international My Home Life (MHL) movement which aims to improve quality of life for care home residents, relatives, and staff [10]. Shared decision-making is an inclusive process where care home staff, the resident and their family make decisions together, using the best available evidence, not only about treatments and care, but about all aspects of life in the care home [11].

Whilst an increased emphasis is being placed on resident choice, control and autonomy in aged care policy reforms internationally [12–14], the key steps to achieving a culture of shared decision-making in the care home setting lack clarity. For the older person living in a care home, having autonomy and being involved in everyday decisions has been consistently highlighted in the research as a determining factor of quality of life [15]. The Mental Capacity Act (2005) in England and Wales [16], and in Northern Ireland (2016) [17] provides a statutory framework that aims to protect and empower people to participate in shared decision-making about their life and care, whilst at the same time ensuring that the individual needs and capacity of each person is taken into consideration. Despite this, evidence suggests that shared decision-making is not the norm and care home staff frequently make decisions on behalf of their residents [18].

Previous research by the authors at Ulster University [5–7] has consistently recommended that older people including individuals moving into a care home, should be empowered to be centre-stage in decisions about their care and support and about their choice of care home. This work by the authors has been acknowledged by the Department of Health (DoH Northern Ireland) who, in 2021, against the backdrop of lessons learned from the COVID 19 pandemic, commissioned the authors to undertake the current literature review. This review forms part of the DoH’s larger agenda to explore how care home residents and their families can be more engaged in decisions about their care. The recent consultation document by the DoH on the Reform of Adult Social Care in N.I. [57] includes the Department’s strategic priority to work with the care home sector in promoting a philosophy of participative decision-making with residents and families to ensure they are involved in all decisions including the operational running of the care home.

Whilst the research literature strongly indicates that shared decision-making benefits residents, families, and staff [11–14], to date there remains a paucity of literature specific to the implementation steps required to successfully achieve shared decision-making in the care home environment. Building and expanding on previous work, this review provides an up-to-date synthesis of shared decision-making in the care home sector and discusses potential enabling and inhibiting factors that influence residents’ and families’ engagement with decision-making about their care and support.

## Aim

The aim was to identify and synthesise literature reporting on the factors that influence (enable and inhibit) care home residents’ and families’ engagement with decision-making about their care and support.

## Methods

An integrative literature review was carried out, guided by the methodological framework proposed by Whittemore and Knafl (2005) and the Preferred Reporting Items for Systematic Meta Analysis (PRISMA) 2020 statement was followed for reporting [20]. An integrative review is a broad research review method that permits quantitative, qualitative, experimental and mixed-method studies to be included, thus facilitating a better understanding of the phenomenon under investigation [21]. Whittemore and Knafl [19] have delineated the process of conducting an integrative research review as encompassing five stages: problem identification stage, literature search stage, data evaluation, data analysis, and presentation. These five stages were used to guide the review process and enhance the rigour of this review.

The problem identification stage was based on a preliminary literature search [19]. At the literature research stage, one researcher (BL) performed a computerised search for peer-reviewed articles in English published from 2011 to 2021. The electronic databases searched included CINAHL, Medline Ovid and ProQuest Health and Medical. These databases were chosen due to the scope of disciplines represented, in conjunction with the wide representation of international journals deemed of relevance for this topic. For the purpose of this review we defined a care home as a residential care facility providing 24-hour care and support for the older person or the person with a disability, who is permanently living there. Additional terms in the search strategy to describe care homes internationally and traditionally were added (nursing homes, residential care homes, residential aged care facilities, long-term care homes).

The search strategy involved defining key words which were refined and grouped within three categories: 1) ‘Care home resident’ OR ‘nursing home resident’ OR long-term care resident’; 2) Families of care home residents’ OR ‘families of nursing home residents’ OR ‘families of long-term care residents’; 3) ‘Decision-making OR ‘shared decision-making’. To capture the relevant literature in care home settings these, search terms were combined with ‘AND’ for: ‘Nursing home’ OR ‘care home” OR ‘long term care’. A full search strategy is provided in more detail [see Additional file 1].

The inclusion and exclusion criteria are detailed in Table 1 below. References from retrieved articles were then searched for additional studies for the final stage of the process.

**Table 1:** Inclusion and exclusion criteria

Inclusion criteria	Exclusion criteria
• Written in the English language. • Studies published in international peer reviewed journals. • Studies inclusive of qualitative, quantitative, and mixed methodologies and case studies. • Systematic literature reviews. • Only studies conducted in care homes or nursing homes or long-term care facilities. • Studies that focus on the perspective of care home residents, families of care home residents and staff in relation to shared decision-making.	• Written in language other than English. • Studies conducted in acute care or transitional care settings. • Studies conducted in the community setting. • Studies prior to 2011 were excluded to give account of existing models and processes. • Studies related to governance and occupational health, as the aim was to explore practice and processes relevant to shared decision-making in care homes

Study abstracts, titles and where necessary, full texts were screened by one researcher (BL) against the inclusion and exclusion criteria and checked by the second researcher (AR). Full texts that matched the inclusion criteria were retrieved and later analysed. During the data evaluation stage, two researchers independently evaluated the papers (BL and AR) using Joanna Briggs Institute Critical Appraisal Tools for qualitative and quantitative studies [62] and the Mixed-Methods Appraisal Tool (2018) for mixed-methods studies [63]. The data analysis stage consisted of data reduction, data display, data comparison, drawing conclusions and verification process [19]. Data were analysed using a thematic synthesis approach as proposed by Thomas and Harden [64]. Based on the research question, BL and AR inductively coded data line-by-line to identify key categories and concepts from the first study. Data from subsequent studies were added to the first, or new concepts and categories where required, in order to develop descriptive themes. Codes and themes were discussed and checked for reliability through continuous peer review with all other researchers (SP and MON). Discussion within the research team helped to resolve any uncertainties or discrepancies.

## Results

**Fig. 1:**
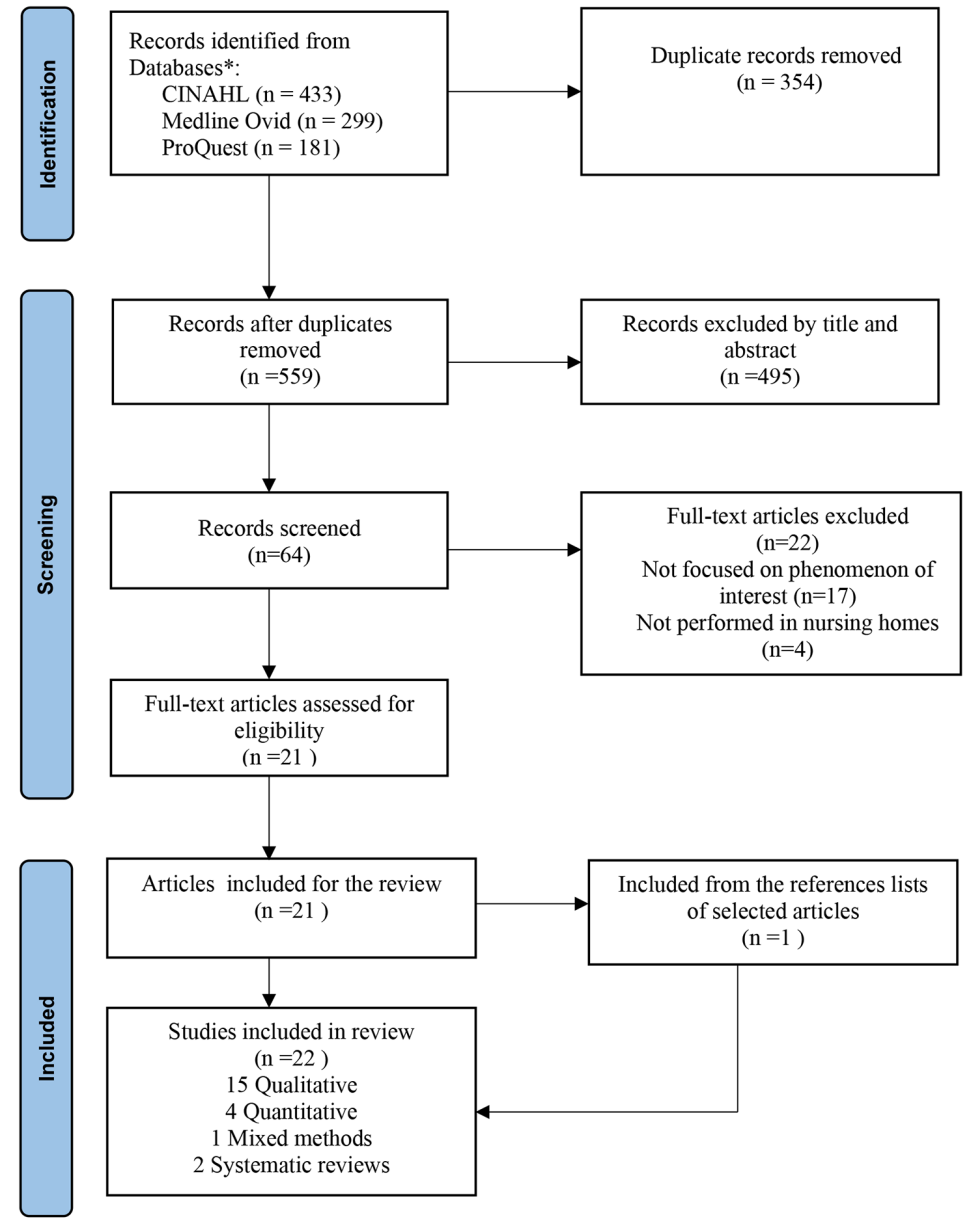
PRISMA flow-chart depicting the main stages of the review process

A systematic search of the databases retrieved 913 results. Duplicates were removed. The initial screening process was undertaken by one researcher (BL) who identified a total 559 articles which were reviewed by title and abstract and 495 found not to be relevant. A total of 64 articles were reviewed fully to assess if they met the inclusion criteria for the review. Further discussion between two researchers enabled consensus to be reached (AR and SP), and finally 21 met our inclusion criteria. Following a manual screening of the reference list of these articles, one additional article was included. A total of 22 papers were deemed eligible for inclusion as they met inclusion criteria previously outline in Table 1. A flow chart showing the number of citations at each stage is described in Fig. 1.

### Synthesis of the included studies

This integrative review included 22 studies conducted in 9 countries, namely, Australia (n = 6), Norway (n = 4), USA (n = 3), Canada (n = 2), Northern Ireland (n = 1), Belgium (n = 2), Spain (n = 1), England (n = 1) and Switzerland (n = 1), with one international study conducted in Italy and the Netherlands. Most of the studies included in the review used qualitative research designs (n = 15), with a combination of in-depth interviews (n = 10) and focus groups (n = 5). The total population for this review, based on the primary studies, was (n = 2,566) and comprised: residents (n = 764), families of residents (n = 1,151), and staff (n = 651). Table 2 below illustrates a summary of the included studies.

**Table 2:** Summary of included studies

Author/Year/Title	Country of Origin	Setting	Methodology & Method	Study Participants	Findings: Enabling, inhibiting & Environmental factors that influence SDM
**Abrahamson et al.** (2016) *“The experiences of family members in the nursing home to hospital transfer decision”* [33]	USA	Nursing Home	Qualitative study Semi-structured interviews	20 Family members	**Inhibiting factors**: Family members felt they were left to make decisions in the absence of clear, accurate information from staff. **Enabling factors**: Recommends development & training for staff to enhance communication skills with families in SDM **Environmental issues**: Family roles are variable depending on their willingness to engage in decision-making.
**Ampe et al.** (2015) *“Advance care planning for nursing home residents with dementia: policy vs. practice”* [28]	Belgium	Nursing Home/ Dementia care units	Quantitative study	Staff from 20 dementia care units	**Inhibiting factors**: ACP conversations with residents & their families on admission, were not ongoing over time. **Enabling factors**: Recommends development of strategies to translate ACP policy into practice. **Environmental issues**: Staff assumed that people with dementia are no longer able to talk about their preferences.
**Arendts et al.** (2015) *“They never talked to me about…’: Perspectives on aged care resident transfer to emergency departments”* [22]	Australia	Residential Aged Care Facility (RACF)	Qualitative descriptive study	6 RAC facilities 14 Family members 17 Staff	**Inhibiting factors**: Low staffing levels, inadequate skill mix & knowledge levels of staff limited staff’s capacity to promote SDM. **Enabling factors**:. Family meetings are seen as a way to include families and improve SDM. **Environmental issues**: Residents are not active participants in the decision-making process & acquiesce as a means of preserving dignity.
**Bauer et al.** (2014) *“Staff–Family relationships in residential aged care facilities: the views of residents’ family members and care staff”* [23]	Australia	Residential Aged Care Facility (RACF)	Qualitative study Family members small group interviews (2–4 participants) or individual interviews. Staff focus groups	5 RAC facilities 14 Family members 27 Staff	**Enabling factors**: Effective communication & sharing mutual information about the resident between staff and families are key factors that promoted SDM. **Inhibiting factors**: Understaffing, staff with English as a second language, non-regular staff, and low education levels, affect staff-family relationships and SDM. **Environmental issues**: Unidentified factors that seem to prevent SDM from being successfully translated into practice.
**Beck et al.** (2017) *“Health care professionals’ perspective of advance care planning for people with dementia in long-term care settings: A narrative review of the literature”* [32]	Northern Ireland	Long-term care(LTC) settings	A narrative literature methodology	205 articles screened from four databases published in English, within time limitation (2002–2014)	**Inhibiting factors**: Lack of recognition by health care professionals (HCPs) that dementia is a condition that would benefit from a palliative approach to care and subsequent initiation of ACP. **Enabling factors**: Education & training for HCPs. Registered nurse has the expertise to see and understand the ultimate consequences of ACP. **Environmental issues**: HCPs perspectives on ACP were influenced by moral & ethical concerns, determining if ACP was initiated or not.
**Bedin et al.** (2013) *“Caring for elders: the role of registered nurses in nursing homes”* [37]	Switzerland	Nursing Home	Qualitative study inspired by activity analysis	9 Nursing Homes 16 Registered Nurses	**Enabling factors**: Expertise of a Registered Nurse (RN) in care homes is a key enabling factor necessary to facilitate SDM. The RN’s attributes of knowing the personhood of the resident & being able to adapt & respond with careful regard to each resident as an individual. **Environmental issues**: Management support & participation are necessary to facilitate the RN’s leadership role in nursing homes.
**Bennett et al.**. (2020) *“Resident perceptions of opportunity for communication and* *contribution to care planning in residential aged care”* [41]	Australia	Residential Aged Care Facility (RACF)	Qualitative inductive study Individual interviews.	6 not-for-profit RAC facilities 102 Residents	**Enabling factors**: SDM is determined by the communication opportunities afforded to the residents and the quality and nature of resident-staff communication during daily care. **Inhibiting factors**: Without communication support many residents experience difficulty expressing preferences & participating in SDM. **Environmental issues**: Lack of reference to communication needs & support in aged care policy & funding assessments, hinders communication support services in RACFs.
**Bollig et al.** (2016) *“They know!—Do they? A qualitative study of residents and relatives views”* [27]	Bergen, Norway	Nursing Home	Qualitative study based on interpretive description Semi-structured in-depth interviews with residents and focus group interviews with relatives	9 Nursing Homes 25 Residents 18 Relatives	**Enabling factors**: A systematic approach to ACP with repeated conversations & support of a key worker for residents & relatives. **Inhibiting factors**: Absence of ACP in nursing homes seems not to be problematic for the residents but may lead to psychological stress for the relatives. **Environmental issues**: Most relatives avoided making important health-related and end-of-life care decisions, deferring these decisions to the knowledge of the staff.
**Cameron, (**2020) *“Challenges faced by* *residential aged care staff* *in decision-making for* *residents with dementia”* [40]	Australia	Residential Aged Care Facility (RACF)	Qualitative exploratory design Individual or group interviews	14 RAC facilities 80 staff	**Inhibiting factors**: Most staff felt that the extent of residents’ participation in SDM should reflect the stage of their dementia. Others suggested that residents cannot generally make decisions at all. **Enabling factors**: Robust policies and procedures on SDM to support staff and a decision-making tool to empower staff. **Environmental issues**: Staff decided from one instance to the next about which of residents’ preferences they should support/ facilitate.
**Cranley et al.** (2020) *“Strategies to facilitate shared decision-making in long-term care”* [24]	Canada	Long-Term Care Home (LTCH)	Qualitative descriptive design Individual semi-structured interviews	40-bed non-for-profit LTCH 3 Residents 3 Family members 3 Staff	**Inhibiting factors**: Residents and families require more emotional support when making difficult decisions on behalf of the resident. **Enabling factors**: Key strategies essential to facilitate SDM in long-term care that include training staff to communicate effectively with residents & families and assigning a key worker for each resident to support the resident in decision-making.
**Fetherstonhaugh et al.** (2016) *“The Red Dress or* *the Blue?” How Do* *Staff Perceive That* *They Support Decision* *Making for People With* *Dementia Living in* *Residential Aged Care* *Facilities?*” [34]	Australia	Residential Aged Care Facility (RACF) Persons with dementia	Qualitative study Individual & group interviews	80 direct care staff	**Enabling factors**: There was an existing culture of relationship-centred care. Staff utilised a range of strategies to support decision-making for the person living with dementia. **Environmental issues**: By limiting the choices offered, staff felt they could preserve the decisional autonomy of the person with dementia whilst also helping to reduce confusion.
**Gjerberg et al.**. (2015) *“End-of-life care communications and shared* *decision-making in Norwegian nursing* *homes - experiences and perspectives of* *patients and relatives”* [29]	Norway	Nursing Home	Qualitative study Semi-structured interviews and focus groups	6 Nursing Homes 35 Residents 33 Relatives	**Inhibiting factors**: Residents & families felt unprepared for SDM. Most patients stated that they had no opportunity to discuss their preferences for treatment and care related to end-of-life. **Enabling factors**: Nursing home staff should take responsibility for initiating conversations about preferences for end-of-life care. SDM should be individualised and iterative. **Environmental issues**: Residents and families acquiesce, deferring decision-making to the staff.
**Godwin (2014)** *“Colour consultation with dementia home residents and staff”* [42]	England	Specialist Dementia Nursing Home	Qualitative study Mixed methods consultation:	1 Specialist Dementia Nursing Home 34 Residents 42 Staff	**Enabling factors**: Visual aids supported communication with people living with dementia, de-emphasising the spoken word and promoting SDM. Result: greater participation and improved self-esteem. **Environmental issues**: Staff helped develop an unpatronising, person-centred approach to SDM.
**Goossens et al.**. (2020) *“Shared decision-making in advance care planning for persons with dementia in nursing homes: a cross-sectional study”* [30]	Belgium	Nursing Home	Quantitative study Cross-sectional design	46 Nursing Homes 311 Staff members 42 Residents	**Enabling factors**: Three-talk model by Elwyn et al. (2012) used to achieve SDM and has utility beyond ACP. **Inhibiting factors**: Staff do not perceive themselves sufficiently competent to practice this guided approach frequently & lack role models in how to apply SDM in their conversations.
**Hanson et al.** (2011) *“Improving decision-making for feeding options in advanced dementia: A randomised, controlled trial”* [38]	USA	Nursing Home	Quantitative study Randomized controlled trial - Questionnaire (Decisional conflict scale administered at 1 and 3 months)	24 Nursing Homes Residents with advanced dementia & families	**Enabling factors: D**ecision aids reduced decisional conflict for families and increased knowledge of treatment options. Family members were more likely to discuss treatments with a healthcare provider, indicating that the decision aid supported rather than replaced clinical communication. The intervention residents were provided with dysphagia diets and experienced less weight loss.
**Helgesen et al. (2015)** *“Relatives’ participation in everyday care in special care units for persons with dementia”* [26]	Norway	Nursing Home- Special Care Unit (SCU)	Quantitative study Study-specific questionnaire (derived from 2 previous studies by same researcher)	23 Nursing Home SCU’s 233 Relatives	**Enabling factors: F**requent visits by family members are valuable for quality of care, as there is a mutual sharing of information between families and staff and this increases residents’ possibility of participating in SDM. **Environmental issues: F**amilies felt decisions about everyday care could be left with staff whom they trusted.
**Mann et al.** (2013) *“Do-not-hospitalize orders for individuals with advanced dementia: Healthcare proxies’ perspectives”* [39]	USA	Nursing Home	Qualitative study Semi-structured interviews	2 Nursing Homes 16 Family members (Health Care Proxies/HCPs)	**Enabling factors**: Families who have had a personal experience in healthcare, an understanding of the prognosis of advanced dementia, and a desire to limit resident distress. **Inhibiting factors**: Families who perceive a lack of physician involvement in decision-making and have a limited understanding of ‘Do-not-hospitalise’ orders and the resident’s prognosis.
**Mariani et al.** (2017) “Shared decision-making in dementia care planning: barriers and facilitators in two European countries” (An Italian and a Dutch LTC setting) [43]	Italy & The Netherlands	Nursing Home	Qualitative study Focus group interviews with HCPs	2 Nursing Homes: 11 HCPs (Italy) 9 HCPs (Netherlands)	**Enabling factors**: Following communication skills training for staff, and specific education sessions for family caregivers, SDM conversations took place between a triad composed of the resident, the family member and a care home professional as the facilitator. Staff scheduled moments during the day to offer residents, together with their family caregivers, an opportunity to express their views and preferences. This approach prompted staff & family to consistently acknowledge the resident’s autonomy and personhood. **Inhibiting factors**: Financial aspects/regulations were key inhibitors.
**Monson et al.** (2021) *“What are the shared decision-making experiences of adult children in regard to their parent/s’ health care in residential aged care facilities?”* [25]	Australia	Residential Aged Care Facility (RACF)	Scoping literature review Mixed methods appraisal tool (version 2011)	597 articles screened from four databases published in English, during period 1985–2019	**Inhibiting factors**: Limited staffing levels & inadequate skill sets of staff inhibit families’ participation in SDM and affect the communication of important information about the resident’s health care. A need for higher educated staff/RNs in RCAF’s in order to promote and engage in SDM with residents and families. **Enabling factors**: In practice, formal SDM and having an equal say are not common.
**Norheim et al.** (2012) *“Factors that influence patient involvement in nursing homes: staff experiences”* [35]	Norway	Nursing Home	Qualitative study Focus group interviews	1 Nursing Home/different wards 16 Multidisciplinary team members	**Enabling factors**: Competence-building programme raised consciousness among staff & changed staff attitudes about person-centred care (PCC) for resident involvement in SDM. **Inhibiting factors**: Time pressure limited PCC and SDM. **Environmental issues**: Lack of sufficient time was considered a key factor that risked generating low-quality care.
**Sarabia-Cobo** (2016) *“Decisions at the end of life made by relatives of institutionalized patients with dementia”* [31]	Spain	Long-Term Care Home (LTCH)	Qualitative study based on naturalistic principles Focus groups Reflective notes by researchers	5 LTC Nursing Homes 84 Family members of residents with dementia	**Inhibiting factors**: Family members’ engagement with decision-making is influenced by their perceptions & feelings about an overwhelming emotional burden & guilt when making decisions on the resident’s behalf; life for the person with dementia is drastically altered by the disease; torn between the two faces of death - the tragedy versus the blessing; reduced quality of life for their loved one; and lack of a specific/key professional to help them understand the processes of the disease.
**Sims-Gould et al., (2014)** *“Autonomy, Choice, Patient-Centered Care, and Hip Protectors: The Experience of Residents and Staff in Long-Term Care”* [36]	Canada	Long-Term Care (LTC)	Qualitative study Focus groups (Part of a larger mixed methods study)	2 LTC facilities 27 Residents 39 Staff	**Inhibiting factors**: Evidence based research and the use of hip protectors as a tool for injury prevention took precedence over resident choice. **Enabling factors**: Training required on safeguarding individual choice and autonomy as well as injury prevention and best practices. **Environmental issues**: Policies are needed to support staff in respecting individual choice even when residents make a choice contrary to what best practice policies might suggest.

A comprehensive review of each study was undertaken to determine enablers and inhibitors to shared decision-making in care homes from the perspectives of staff, residents, and families, alongside environmental issues. The thematic analysis identified six key factors that enable shared decision-making, four key factors that inhibit shared decision-making and four key environmental issues. These were subsequently categorised into three main themes that illustrate the complexities of shared decision-making in care homes (Table 3).

**Table 3:** Overview of Themes

Themes	Enabling factors	Inhibiting factors	Environmental issues
**A positive culture of collaborative and reciprocal relationships**	Trust and communication Existing culture of relationship-centred care Role of the Registered Nurse: knowing the personhood of the resident & being able to adapt & respond with careful regard to each resident as an individual.	Time pressure, low staffing levels, inadequate skill mix and knowledge levels of staff	Management support and participation are necessary conditions for SDM
**Willingness to engage and a willingness to become engaged**	Suggested strategies to facilitate SDM: for example, developing the skills of staff in relation to SDM, and assigning a key worker for each resident to support SDM	Lack of competence & confidence by staff in how to apply SDM in their conversations Residents and families’ unpreparedness for SDM	Staff assume that people with dementia are no longer able to talk about their preferences. Residents & families acquiesce, deferring decision-making to the staff
**Communicating with intent to share and support rather than inform and direct**	Balancing an appropriate level of independence with an appropriate level of risk Seeing decision-making as a supportive process rather than a once off event	Paternalistic practices of staff	Top-down approach versus a bottom-up approach

*SDM* shared decision making

The three themes are:

- A positive culture of collaborative and reciprocal relationships.
- A willingness to engage and a willingness to become engaged.
- Communicating with intent to share and support rather than inform and direct.

### A positive culture of collaborative and reciprocal relationships

#### Trust and communication

Supporting and nurturing collaborative relationships between residents, families, and staff is an essential component of shared decision-making in care homes. Evidence from the review shows that the notion of trust and communication is seen as being central to collaborative relationships [22–25]. Bauer et al. [23] explored the perceptions of staff (n = 27) and family members (n = 14) in relation to each other’s roles and responsibilities in an Australian residential aged care setting. The findings revealed that being listened to and valued was identified by families as central to a trusting relationship with staff. Evidence in the literature review supports this view, highlighting that a relationship-centred approach plays a significant role in family members’ ability to participate in shared decision-making [23–25]. Bauer et al. [23] found that constructive relationships between staff and families were seen to develop when there was a reciprocal sharing of information about the resident’s care. Families expressed that when they shared their knowledge about their relative with the staff, this was respected and utilised by the staff to deliver care in a person-centred way. Equally, staff described that they trusted the families’ observations when they reported subtle changes in their relatives’ condition. Some families, however, also reported that collaborative relationships and the development of trust between staff and families were hampered because staff from other cultures did not have English as their first language. Management support and participation is a key environmental issue identified throughout the review. Many families suggested that management support and participation were necessary conditions for shared decision-making in the care homes and they also expressed the need for knowledgeable and dependable staff who consistently provided quality care [25, 26, 35].

In Norway, Helgesen et al. [26] explored relatives’ participation in everyday care in special care units for persons living with dementia and reported contradictory results. Most relatives felt their point of view was listened to by staff and that staff supported them in their role as relatives. However, relatives also reported that they seldom participated in decisions concerning the resident’s everyday care, feeling that these decisions could be made by the staff whom they saw as having the knowledge and whom they trusted. In contrast, relatives in the study by Bollig et al. [27] reported that Advance Care Planning (ACP) and shared decision-making is lacking in nursing homes and many family members are unaware of their relative’s wishes in relation to their life in a care home. Most relatives felt that it was too difficult to make decisions on behalf of the resident, especially in relation to health-related and end-of-life care decisions. They therefore avoided making important decisions, deferring these to the expertise of the staff. The evidence presented in the literature reveals that whilst the absence of ACP may not seem to be problematic for the resident, it can and often does lead to psychological stress for the relatives [27, 32].

Seven articles focused on advance care planning (ACP) or end-of-life perspectives of residents, families, and staff [27–32]. One study found that whilst there was an ACP policy in place, and staff did involve residents with dementia and their families in the initial admission conversation, ACP conversations were not ongoing and did not accommodate or allow for a change in the person’s original decision [28]. Two studies found a lack of information provision to residents and their families and reported that staff rarely discussed the risks and benefits of treatment options [28, 33]. In a narrative review of the literature, Beck et al. [32] found evidence that dementia remains unrecognised by many staff working in long-term care settings as a condition requiring palliation. Staff perceived communication difficulties with both the person with dementia and their families and viewed this difficulty as an inhibiting factor to the initiation of shared decision-making in the long-term care setting.

#### Existing culture of relationship-centred care

Nine of the studies analysed concepts related to a relationship-centred or person-centred approach to care [22–25, 27, 33–35, 37]. In two of the studies, an existing relationship-centred culture was highlighted by staff as being a key enabler to shared decision-making and family involvement [34, 35]. Findings from other studies included in the review illustrated that where a relationship-centred approach to care was not fully embraced, or embraced only in rhetoric, staff adopted various tactics that resulted in a lack of shared decision-making [22, 34]. Time pressure, inadequate skill mix, inadequate knowledge levels of staff, and routine linked to task orientated care were key inhibiting factors to promoting meaningful staff-resident and family relationships and shared decision-making [22, 23, 25, 32].

#### Role of the registered nurse

The literature review identified three papers highlighting the importance of the expertise of the Registered Nurse (RN) in nursing homes [25, 32, 37]. This was identified as being a key enabling factor necessary for shared decision-making [35]. The expertise in the nursing role comprises diagnostic, therapeutic, and ethical judgment, and acting in the best interests of the person who is receiving care. Evidence shows that the registered nurse is the central axis of the multiple relationships that she/he must maintain with other staff, the residents, and their relatives [1]. The aim of this web of relationships is to share decision-making. Bedin et al. [37] describe the nursing role as *“an indispensable linchpin”* that is essential to the consistency of a person-centred perspective, and to *“maintaining professional values in situations of ethical tension”* (p.117). The study emphasised that management support and participation are necessary conditions for the registered nurse’s leadership mission in the care home sector to be fulfilled. Research by Phelan and McCormack [1] which explored the expertise of registered nurses in residential care for older people in the Republic of Ireland, supports these findings. This empirical research revealed several important attributes that represent nursing expertise in residential care of older people, among them are knowing the personhood of the resident and being able to adapt and respond with careful regard to each resident as an individual. A key finding from the research indicates how nurses, in their day-to-day practice, demonstrate their expertise by assessing up-to-date evidence and evaluating the quality of that evidence to ensure it is appropriate for the individual resident and their condition. The related conversations the nurse has with the resident based on their assessment, interpretation and application of evidence is a crucial factor in enhancing the ability of the residents (and that of their family) to make decisions on their own behalf. In a culture of relationship-centredness, shared decision-making utilises evidence to provide the resident with various options to support their decisions, as opposed to coercing residents into practices/treatments that conflict with their preferences and values. Whilst both studies have demonstrated the uniqueness of nursing expertise in relation to shared decision-making in the care home setting [1, 37] they both recommend the necessity of further research that will help elucidate the capabilities of nurses in this setting.

### Willingness to engage and a willingness to become engaged

#### Acquiescence: deferring decision-making to the staff

The degree to which family members were willing or ready to engage in decision-making was variable within and across studies, particularly in relation to ACP and end-of-life care [26, 28, 29, 39, 40]. In five of the studies, resident and family willingness to become engaged was shown to be a significant enabling factor in the process of shared decision-making. Whilst most relatives and a number of residents expressed a desire to engage in end-of-life conversations, many residents did not find it essential to have these conversations or were more reluctant to talk about these issues. The literature points to multi-factorial reasons for the low incidence of such conversations. Arendts et al. [22] found that residents as a group are least likely to be active participants in the decision-making process and are more likely to acquiesce as a means of preserving dignity. A study by Abrahamson et al. [33] in the USA, explored family members’ experience in relation to hospital transfer decisions for the resident and identified that family roles are variable depending on their willingness to engage in decision-making. Most family members’ perceptions were that staff did not address changes in the resident’s condition promptly enough to avoid hospitalisation. These families believed that staff should have identified and communicated a need for hospitalisation earlier. However, families also reported that because the staff knew their resident and their needs so well that this was a key benefit of remaining in the care home and avoiding hospitalisation.

In the study by Helgesen et al. [26], there were 233 family members of persons with dementia surveyed. Most of them reported that they did not participate in decision-making, nor did they express a desire to do so. This was even though half of the families in the study saw their participation as being crucial for person-centred care. With respect to end-of-life care conversations, Gjerberg et al. [29] reported that some residents and families expressed unpreparedness for shared decision-making and wanted to leave the decisions more or less completely to the nursing home staff. Findings from a Spanish study [31] revealed that most family members (n = 84) of persons living with dementia expressed an enormous emotional burden and a strong sense of guilt in their role as decision-maker for their relative. Families indicated that having a key member of staff to provide informational and emotional support would have helped with decision-making and eased the burden. Willingness of families to become engaged in shared decision-making was assessed in one study [28] by staff asking the families about their preferred approach to receiving information to assist in their decision-making: for example, by discussing various issues with a staff member in one-to-one consultations, receiving printed material and/or receiving information through the use videotapes or other media. Despite this, another key environmental issue revealed in the study was that staff generally talked about preferences with the families instead of the residents and assumed that people with dementia were no longer able to articulate their preferences. Mariani et al. [43] emphasise that whilst there is a deterioration in the person’s abilities to answer fact-based questions after the early stages of dementia, their abilities to answer preference questions remain more stable over time. Studies in the literature reviewed indicate that it is possible to assess the personal preferences of people with dementia and enhance their decision-making involvement [32, 34]. The lack of skills by staff to recognise and facilitate the resident’s desire and ability to decide is a significant and consistent inhibiting factor throughout the literature reviewed [22, 30, 32, 36, 41, 43].

Two studies highlighted staff’s feelings of ‘uneasiness’ and a sense of ‘discomfort’ towards discussing ACP or end-of-life issues which resulted in a general hesitancy by staff to engage in shared decision-making [28, 32]. Suggested possible reasons for this discomfort was a reluctance by staff to discuss death and a fear of upsetting the people in their care. Gjerberg et al. [29] found that most residents and families wanted conversations about end-of-life care. However, if such conversations were not initiated by the staff, then the residents’ and families’ needs remained unmet.

The willingness of staff to engage residents in shared decision-making is largely determined by resident-staff communication during day-to-day care. It is evident from the literature reviewed that in the absence of communication support, many residents will continue to have trouble expressing preferences about their care and participating in shared decision-making [29, 41, 42]. Findings from a Norwegian study [29] recommend that nursing home staff should take responsibility for initiating conversations about preferences for end-of-life care, assisting and supporting residents to talk about these issues, while at the same time being sensitive to the diversity in opinions and the timing of such conversations. Most residents stated there was no opportunity to discuss their values and preferences for treatment and care related to end-of-life with the nursing home staff. Some explicitly said that they wanted or missed this kind of conversation.

#### Suggested strategies to facilitate shared decision-making

Whilst education and training in shared decision-making was not a focus of the literature reviewed, many of the articles suggested strategies to improve communication and cooperation between staff and families and to facilitate shared decision-making [28, 33, 38]. A study by Mariani and colleagues [43] explored the barriers and facilitators regarding the implementation of a shared decision-making framework for care planning in two nursing homes, one in Italy and one in the Netherlands. The researchers adopted the philosophy that shared decision-making was an opportunity for people with dementia to express their opinion and wishes during the care planning process. Following communication skills training for staff, shared decision-making conversations took place between a triad composed of the resident, the family member, and a care home professional as the facilitator. The role of the family caregiver was to support and facilitate the resident’s expression of opinion during the conversations. The findings indicated that communication skills training is an essential prerequisite for implementing shared decision-making in dementia care. Staff scheduled moments during their daily practice to ask the residents direct questions about their wishes, and offered residents, together with their family caregivers, an opportunity to express their views and preferences. This approach prompted staff and family caregivers to become aware of, and acknowledge, residents’ autonomy and personhood. The contribution of the nursing home managers in the implementation of shared decision-making was seen as essential, not only to the accomplishment of the primary objectives of the intervention but also to the improvement of other secondary aspects. Several articles in the review echo the importance of the care home manager’s contribution and role in shared decision-making [24, 37].

Similarly, following a competence-building programme for staff, Norheim et al. [35] reported that the programme had raised consciousness among staff and influenced a change in staff attitudes. The emphasis in the culture of care had moved from a focus on tasks and routines to a more person-centred focus. Along with highlighting the importance of providing staff with training and developing their skills in shared decision-making, several studies also recognised the importance of assigning a key worker for each resident to support the resident in decision-making and facilitate open, proactive communication with the resident, their family and the staff [24, 35, 40].

### Communicating with intent to share and support rather than inform and direct

#### Balancing an appropriate level of independence with an appropriate level of risk

Several studies demonstrated contrasting approaches to utilising evidence with the intention of facilitating shared decision-making. The review identified a top-down approach versus a bottom-up approach by staff as one of the environmental issues. Sims-Gould et al. [36] describe a top-down approach where staff held the knowledge of best practice and used it to inform and direct residents in the use of hip protectors as a tool for injury prevention. This belief was so deeply engrained in staff care practices that in some instances, staff would insist on the use of hip protectors even when residents had explicitly declined their use. The residents’ choice and autonomy were strongly denied. In relation to residents’ perspectives concerning transfers to emergency departments, Arendts et al. [22] found that shared decision-making and meaningful engagement rarely occurred. In some instances, staff adopted a paternalistic attitude, denying residents’ and relatives’ choice. Paternalistic attitudes are frequently driven by the staff’s belief that they know what is best for residents. However, there is also evidence that care home staff experience moral distress when they feel they must transfer a resident to acute care (at the behest of relatives), even though they know the residents would prefer to stay in the care home environment [44]. Communicating with intent to share and support is contingent on finding the right balance between introducing evidence-based practice and ensuring the approach to utilising evidence does not take precedence over residents’ preferences and values.

Communicating with intent to share and support is perceived as a bottom-up approach where residents and families are provided with evidence of treatment risks and benefits. In the study by Hanson et al. [38], a bottom-up approach was used, in which a decision aid about treatment (feeding) options in advanced dementia was effective in improving the quality of decision-making by families of nursing home residents with dementia. Family members were more likely to discuss treatments with staff, indicating that the decision aid supported rather than replaced communication.

#### Seeing decision-making as a supportive process rather than a once off event

In exploring the challenges faced by staff in decision-making for residents living with dementia, Cameron [40] reported that most staff suggested that residents can only make decisions on matters where the consequences carry very little risk or that they cannot generally make decisions at all. Without support around decision-making for residents with dementia, staff felt they had the added burden of having to decide from one instance to the next about which of the resident’s preferences they should support or facilitate.

There is evidence in the literature of models that use a guided, staged approach to achieve shared decision-making. Decision-making is viewed as a supportive process rather than a once off event [28, 30, 34, 38]. In its simplest terms, the process involves, providing people with choices, then narrowing those choices down to options and then making a decision. One study used ‘The three-talk model’ [30] as a guided approach to achieve shared decision-making during ACP conversations with persons with dementia. It consists of introducing options (choice talk), discussing these options (option talk), and then making a decision after exploring preferences (decision talk). It provides a practical, easy way to skill up clinicians in shared decision-making and has utility beyond ACP.

Fetherstonhaugh et al. [34] reported that there was an existing culture of person-centred care in the care homes recruited to their study, and staff had an awareness of strategies to support resident decision-making. These strategies had the aim of simplifying the process of decision-making for the person with dementia. For example, when staff members helped the person to get dressed, rather than opening the wardrobe to show the residents all the clothing on display, causing them to become overwhelmed, they reduced the number of options for the resident to choose from. This encouraged the resident to make a decision and avoided them becoming confused or upset.

Communicating with intent to share and support was clearly demonstrated in Godwin’s study [42]. The study illustrated the communication opportunities that staff afforded residents living with dementia in an extended care environment. In consulting the residents about the care home décor, staff helped to develop an unpatronising, person-centred approach to shared decision-making which minimised the need for speech for residents with communication difficulties. Visual aids helped in seeking the residents’ opinions and choice. The approach supported communication, deemphasised the spoken word and promoted inclusion. The researcher noted that the residents appeared to be ‘surprised and pleased’ to be asked their opinion and to be included in decision-making (p.114). Godwin argues that this kind of consultation could enhance the self-esteem of persons with dementia and contribute to their quality of life. The findings reveal that the use of visual aids, observation, activity, and non-verbal communication achieved higher than expected levels of participation from the residents.

## Discussion

This integrative review aimed to explore the enabling, inhibiting, and environmental factors that influence care home residents’ and families’ engagement with decision-making about their care and support. The themes outlined in the review illuminate the complexities involved in engaging residents and their families in shared decision-making. The key findings of the review reveal several factors that significantly impact shared decision-making in care homes. This section of the report places these key findings in the context of the existing evidence base and are discussed under the following headings: skill mix and turnover; competent and confident staff; the older person’s position in the decision-making loop; and potential for innovative risk taking.

### Skill mix and turnover

The review highlights a number of strategies to facilitate shared decision-making including the importance of assigning a key worker for each resident. The assignment of a key worker to support residents and their relatives in decision-making is contingent on staffing levels and staff turnover rates in the care home. Skills for Care (2020) reported that the average turnover rate of care homes in the UK was 26% in 2016/17, rising to over 30.4% in 2019/20 [45]. In their study exploring the views and experiences of residents and staff in care homes, Ryan and Moore (2021) [46] found that a high turnover of staff can significantly impede the promotion of relationship-centred care and residents’ autonomy. Their findings identified that when only one person was skilled up in a particular area and resigned their post, for example the activities co-ordinator, activities and social outings for the residents were cancelled. Not only was the care home depleted of that resource but due to the high turnover and staffing shortages, staff were left prioritising the physical needs of residents over their social and psychological care.

Public perceptions of care homes along with a constant stream of bad news in the media, present major challenges for care homes in recruiting staff with the knowledge, skills and attitudes to care for some of the frailest and most vulnerable people in society. Findings from the study by Thompson et al., (2015) [47] revealed several factors underlying the difficulties with recruitment and retention of nursing staff in care homes. Nurses reported feeling stigmatised by their NHS colleagues due to the perceived low status nature of their work. The ageist attitude of some healthcare professionals was found to contribute to the perception that nurses caring for older people are less skilled than other nurses. Nurses were also concerned that privately owned care homes prioritised profit over people, leading to high levels of moral distress among staff. Whilst a primary difficulty faced by nurses was the tension between care and funding, many nurses saw a key part of their role as supporting residents in making decisions regarding their transition to the care home. Nurses felt that an essential part of the shared decision-making process involved showing potential residents around the home and discussing how the home could best meet their specific needs [47].

A key finding of the review was the importance of the role of the Registered Nurse in relation to shared decision-making. Several important attributes of the Registered Nurse were identified as being fundamental to engaging residents and families in decision-making about their care and support. These include knowing the personhood of the resident and being able to adapt and respond with careful regard to each resident as an individual. The reality, however, is that very often there is only one nurse on duty in a care home at any one time with responsibility for managing the care home and ensuring safe and effective care provision for all residents [1, 48]. Managing skill mix and staff shortages, along with the increasing pressure from regulatory requirements and reporting mechanisms, all impose time constraints that impede the Registered Nurse’s engagement in shared decision-making. Studies have shown that establishing relationships with residents and facilitating shared decision-making is not only a core nursing function but one that also influences retention of Registered Nurses in the care home sector [49, 50]. Although nurses want to nurture relationships and engagement with residents and their families, the incentive can be undermined by challenging organisational systems and a heavily regulated care environment. Shared decision-making may therefore be perceived by nursing staff as a luxury they can only undertake when they have time.

In the Netherlands, Koopman at al., [51] found that a more diverse staff skill mix with a higher percentage of professional nurses had a positive effect on quality of care and quality of life for residents living in residential care homes. Empirical research by Aiken et al., [52] has clearly shown that a higher Registered Nurse skill mix in hospitals is associated with a significant reduction in mortality and morbidity rates, higher safety, and higher patient ratings of their care. This is a crucial finding and whilst it is located in the hospital setting, it could be argued that the outcomes could be similar in care homes. Indeed, these findings can be seen as an initial step in gaining insight into how an increase in the Registered Nurse skill mix could help achieve a culture of shared decision-making in the care home setting. Time pressure, low staffing levels, inadequate skill mix and knowledge levels of staff are identified in the review as major inhibitors to shared decision-making. It is essential that an urgent focus is placed on these inhibiting factors in order to get to the point where shared decision-making becomes embedded in the culture of the care home sector.

### Competent and confident staff

Other strategies evidenced in the review included developing the skills of staff in relation to shared decision-making. Whilst this is a noble suggestion, the care home sector has a poor history of supporting and developing staff compared to, for example, their counterparts in the NHS [53]. Indeed, training and education requirements for care assistants in long-term care facilities in many European countries are low and very few countries in the Organisation for Economic Co-operation and Development (OECD) have developed a career structure for staff working in care homes [54]. In most countries, almost anyone can become a personal care worker. Less than half of the surveyed countries in the OECD require that personal care workers hold a minimum education level. Colombo et al. [55] found that in most OECD countries, fewer than half the staff working in long term-care are nurses. Challenging working conditions and low pay often generate a high turnover of staff, contributing to a negative image of the sector, which in turn can threaten both access to, and quality of, services.

Several studies in the review identified that limited knowledge levels of staff had an inhibiting influence on residents’ and families’ participation in shared decision-making [22, 23, 25]. Ensuring that staff are trained and supported is primarily the responsibility of the care home provider. Financial pressures coupled with safe staffing levels are key challenges for providers and often take precedence over the provision of staff training and development [61]. Whilst providers ensure that staff receive essential mandatory training, the current funding model for long-term care does not provide for the ongoing professional development of care home staff [56]. It is therefore unrealistic to expect care home staff to automatically have the skills to engage residents and their families with decision-making. A serious commitment to promoting shared decision-making will require exploring how the sector can be adequately resourced to create a culture where staff are competent and confident in facilitating shared decision-making. That said, the recent consultation document by the Department of Health (Northern Ireland) on the Reform of Adult Social Care in N.I. (Jan 2022 [57], includes proposals to develop career pathways, supervision and support, training and education of the care home workforce and to raise the profile and recognition of the sector. The document also highlights the Department of Health’s plan to work with the care home sector in promoting a philosophy of participative decision-making with residents and families to ensure they are involved in all decisions including the operational running of the care home.

### The older person’s position in the decision-making loop

The degree to which family members were willing or ready to engage in decision-making was variable within and across studies, particularly in relation to conversations about ACP and end-of-life care for their relative. Evidence from several studies indicated that while residents were reluctant to talk about ACP and end-of-life care, many residents were not afforded an opportunity to discuss their preferences about these issues with the care home staff [29, 32, 40]. This passive approach by residents may be influenced by their experience of consistently being outside the decision-making loop throughout their care trajectory. A recent study by Combes et al. (2021) found that frail older people receiving a community-based older persons’ service run by a large urban UK hospice, did not see the relevance of advance care planning for themselves, either because they did not believe they were ill enough to engage or because they preferred to focus on living well now [65]. In Arendt’s et al’s study [22], most residents reported that they were resigned to being transferred to the emergency department (ED) from their care home because the decision would be made by someone else. Some residents who expressed a desire for their views to be heard, reported having no real expectation that they would be. Ageism and paternalistic attitudes still permeate service provision to older people and the reality is that most older people are y not involved in decisions about their care and support [57]. In relation to the older person transitioning to a care home, O’Neill et al. (2020c) [58] found that most older people were not involved in the decision-making process, nor did they have a choice in selecting the care home. Whilst most of the residents in the study had the capacity to make autonomous decisions, health and social care professionals and family members had a stronger influence on all aspects of decision-making. [46]. The older people interviewed also reported that covert decisions were often made by hospital staff in collusion with families about the need for a care homes placement. It is therefore understandable that because of the way their move to the care home was managed, residents felt very sceptical and lacked trust in the system. Consequently, many residents found it difficult to feel empowered in making decisions after they moved into the care home [46, 59]. The care home environment itself and the need to comply with regulations, can be perceived by residents as imposing limitations on their decision-making. When the impact of regulations mean that residents often feel restricted from dining alone in their room (risk of choking) and moving freely (risk of falling) in the care home, it is easy to understand how they can become disempowered in relation to their decision-making abilities.

### Potential for innovative risk taking

The difficulty for staff in balancing an appropriate level of independence for the resident with an appropriate level of risk was evident in the review as a potential inhibitor to shared decision-making. Whilst this imbalance may manifest as a paternalistic approach to care, the impact of a heavily regulated care home environment limits the autonomy of the Registered Nurse to use their professional judgement and take innovative risks [6, 50] Although, the implementation of these regulations were developed to enhance care, they are perceived by staff as institutionalising the care home. The many regulations and strict risk management policies may not only constrain nurses’ creative thinking and professional judgement but also may threaten the resident’s autonomy and decision-making. In terms of the regulator, therefore, there is no medium whereby a decision can be made about the risks that residents and their families are comfortable in taking. This rule-driven environment with an emphasis on standards as opposed to the promotion of human rights and autonomy, can make it difficult for staff to model shared decision-making. Providers, managers and the regulator need to work together to develop the service in order to protect and promote the human rights of care home residents. Part of this work should also include empowering care home staff to formulate ways to engage residents and families in decisions about risks while also supporting staff to stay within the limits of regulation.

My Home Life (MHL), previously referred to in this paper, is an international programme that aims to promote quality of life and positive change in care homes. The programme offers leadership support to provide managers with the knowledge and skills to inspire and lead culture change in care homes [60]. MHL (www.myhomelife.org.uk) is guided by eight best practice themes along with the evidence base of relationship-centred care [8]. Shared decision-making is one of the eight themes and considered the pinnacle of relationship-centred care [8, 9]. Within the MHL network, a number of different initiatives have been undertaken by care home managers that facilitate shared decision-making. One such example is a ‘Decision Tree’ which is used to facilitate the involvement of residents in decisions about life in their care home. A small artificial tree is kept in the lobby and the residents use luggage tags to write their suggestions (for example, where to go to for their next outing) before placing them on the tree. The approach provides alternative communication opportunities, particularly for residents who may feel uncomfortable speaking out in a group. Other examples can be seen on https://www.myhomelifeni.co.uk/.

**Key Recommendations**.

1. There is a need to undertake an evaluation study to explore the shared decision-making experiences of care home residents, their families, and staff about their care and support. This will provide a baseline for future shared decision-making implementation processes in the care home sector.
2. Guided approaches and strategies evidenced in the review could be further co-developed with care home staff, residents, and their families, and tested and implemented to facilitate shared decision-making.
3. There is a need to provide staff with education and training to enable the implementation of shared decision-making in care homes.
4. There remains a lack of literature specific to the implementation steps required to successfully achieve shared decision-making in the care home environment. Therefore, further research needs to be undertaken to explore how shared decision-making can be better facilitated in care homes.
5. Despite evidence of shared decision-making in care homes across the MHL network, there is currently no clear mechanism in place to disseminate this good practice. Care home staff should be encouraged to publish their work and to present at conferences and seminars. Dissemination of this work is important in order to provide evidence of the research impact on culture and practice across the care home sector.

## Conclusion

This review points to the complex factors that influence care home residents’ and families’ engagement with decision-making. One of the most important findings emerging from the review is that the implementation of shared decision-making in care homes is highly dependent on the support and nurturing of collaborative and reciprocal relationships between residents, families, and staff. Part of this process includes ascertaining the willingness of residents and families to become engaged in shared decision-making. The review highlights the importance of finding the right balance between introducing evidence-based practice and ensuring the approach to utilising evidence does not take precedence over residents’ preferences and values. Communication skills training for staff and guided approaches that view decision-making as a supportive process rather than a once off event are essential prerequisites for implementation.

There remains a lack of literature specific to the implementation steps required to successfully achieve shared decision-making in the care home environment. Therefore, further research needs to be undertaken to explore how shared decision-making can be better facilitated in care homes.

## Electronic supplementary material

Below is the link to the electronic supplementary material.

Supplementary file 1. Appendix 1: Literature search strategies

## Data Availability

All data generated or analysed during this study are included in this published article.

## List of abbreviations

ACP: Advance Care Planning.
LTCH: Long Term Care Home.
MHL: My Home Life.
PCC: person-centred care.
PRISMA 2020 statement: Preferred Reporting Items for Systematic Meta Analysis 2020 statement.
RACF: Residential Aged Care Facility.
RN: Registered Nurse.
SCU: Special Care Unit.
SDM: shared decision-making.
