# Supplementary material for: The factors that influence care home residents’ and families’ engagement with decision-making about their care and support: an integrative review of the literature
Source: BMC Geriatr. 2022 Nov 17;22:873. doi: 10.1186/s12877-022-03503-8 (PMC9672635; doi:10.1186/s12877-022-03503-8)
Supplement: Supplementary file 1 — Appendix 1: Literature search strategies [file 12877_2022_3503_MOESM1_ESM.docx]

**Appendix 1: Literature search strategies**

1. **EBSCO CINAHL** (Searched 20^th^ July 2021)

| **Search ID** | **Search Terms** | **Search Options** | **Actions** |
| --- | --- | --- | --- |
| S8 | S5 AND S6 | Limiters - Published Date: 2011-2021; Language: English; Age Groups: All Adult | 433 |
|  |  | Expanders - Apply equivalent subjects  Search modes - Boolean/Phrase |  |
| S7 | S5 AND S6 | Expanders - Apply equivalent subjects  Search modes - Boolean/Phrase | 976 |
| S6 | S3 OR S4 | Expanders - Apply equivalent subjects  Search modes - Boolean/Phrase | 141,362 |
| S5 | S1 OR S2 | Expanders - Apply equivalent subjects  Search modes - Boolean/Phrase | 17,897 |
| S4 | "shared decision making" OR "shared decision - making" | Expanders - Apply equivalent subjects  Search modes - Boolean/Phrase | 6,233 |
| S3 | (MH "Decision Making+") OR (MH "Decision Making, Shared") OR (MH "Decision Making, Patient+") OR (MH "Decision Making, Family") OR (MH "Decision Making, Ethical") OR (MH "Decision Making, Clinical+") | Expanders - Apply equivalent subjects  Search modes - Boolean/Phrase | 138,838 |
| S2 | "care home residen*" or "nursing home residen*" or "residential care" or "nursing home care" or "long term care" or "famil* of nursing home residen*" or "famil* of care home residen*" or "famil* of long term care residen*" or "informal carer of care home residen*" or "informal carer of nursing home residen*" or "informal carer of long term care residen*" | Expanders - Apply equivalent subjects  Search modes - Boolean/Phrase | 6,888 |
| S1 | MH "Nursing Home Patients" OR MH "Nursing Homes" | Expanders - Apply equivalent subjects  Search modes - Boolean/Phrase | 14,764 |

1. **MEDLINE (Ovid)** (Searched 20^th^ July 2021)

| **Search**  **number** | **Search terms** | **Results** |
| --- | --- | --- |
| 1 | exp Nursing Homes/ | 42,370 |
| 2 | exp Caregivers/ | 43,896 |
| 3 | ("care home residen*" or "nursing home residen*" or "long term care residen*" or "famil* of care home residen*" or "famil* of nursing home residen*" or "famil* of long term care residen*" or "informal carer of care home residen*" or "informal carer of nursing home residen*" or "informal carer of long term care residen*") | 9,127 |
| 4 | exp Decision Making, Shared/ | 1,393 |
| 5 | ("decision making" or "shared decision making" or " shared decision-making") | 245,328 |
| 6 | 1 or 2 | 87,584 |
| 7 | 3 or 4 | 245,328 |
| 8 | 5 or 6 | 3,666 |
| 9 | 6 and 7 and 8 | 1,212 |
| 10 | Limit 9 to (english and journal article and last 10 years) | 299 |

1. **ProQuest Health & Medical Collection** (Searched 20^th^ July 2021)

| **Query** | **Items** |
| --- | --- |
| (“decision-making" OR “shared decision-making" OR “shared decision making”) AND ("care home residen*" OR "nursing home residen*" OR "famil* of nursing home residen*" OR "famil* of care home residen*" OR "famil* of long term care residen*" OR "informal carer of care home residen*" OR "informal carer of nursing home residen*" OR "informal carer of long term care residen*") AND ("residential care" OR "nursing home care" OR "long term care") AND stype.exact("Scholarly Journals") AND la.exact("English") | 181 |
